# Gas-phase intermolecular phosphate transfer within a phosphohistidine phosphopeptide dimer

**DOI:** 10.1016/j.ijms.2014.04.015

**Published:** 2014-06-15

**Authors:** Maria-Belen Gonzalez-Sanchez, Francesco Lanucara, Gemma E. Hardman, Claire E. Eyers

**Affiliations:** a Michael Barber Centre for Mass Spectrometry, School of Chemistry, Manchester Institute of Biotechnology, University of Manchester, 131 Princess Street, Manchester M1 7DN, UK; b Institute of Integrative Biology, University of Liverpool, Crown Street, Liverpool L69 7ZB, UK

**Keywords:** NCX, non-covalent complex, pArg, phosphoarginine, pHis, phosphohistidine, pLys, phospholysine, TWIMS, travelling wave ion mobility-mass spectrometry, CID, Phosphotransfer, Phosphoramidate, Histidine phosphorylation, Gas-phase dimer, Non-covalent interactions

## Abstract

The hydrogen bonds and electrostatic interactions that form between the protonated side chain of a basic residue and the negatively charged phosphate of a phosphopeptide can play crucial roles in governing their dissociation pathways under low-energy collision-induced dissociation (CID). Understanding how phosphoramidate (i.e. phosphohistidine, phospholysine and phosphoarginine), rather than phosphomonoester-containing peptides behave during CID is paramount in investigation of these problematic species by tandem mass spectrometry. To this end, a synthetic peptide containing either phosphohistidine (pHis) or phospholysine (pLys) was analyzed by ESI-MS using a Paul-type ion trap (AmaZon, Bruker) and by traveling wave ion mobility-mass spectrometry (Synapt G2-S*i*, Waters). Analysis of the products of low-energy CID demonstrated formation of a doubly ‘phosphorylated’ product ion arising from intermolecular gas-phase phosphate transfer within a phosphopeptide dimer. The results are explained by the formation of a homodimeric phosphohistidine (pHis) peptide non-covalent complex (NCX), likely stabilized by the electrostatic interaction between the pHis phosphate group and the protonated *C*-terminal lysine residue of the peptide. To the best of our knowledge this is the first report of intermolecular gas-phase phosphate transfer from one phosphopeptide to another, leading to a doubly phosphorylated peptide product ion.

## Highlights

• Fragmentation of phosphoramidate-containing peptides by CID in a QIT was assessed.
• pHis homodimer formation facilitates intermolecular phosphate transfer during CID.
• Dimer formation and phosphate transfer is dependent on a *C*-terminal basic Lys.

## Graphical abstract

**Figure:**
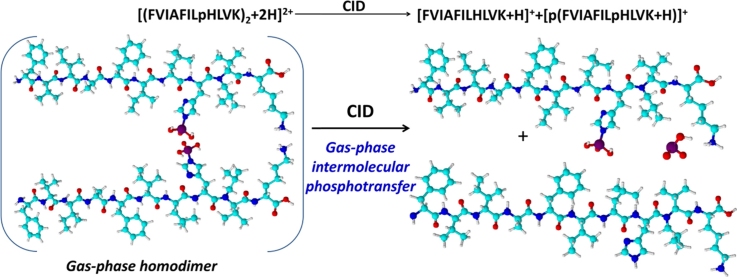

## 1 Introduction

Mass spectrometry has become an extremely powerful analytical tool for the qualitative and quantitative analysis of protein phosphorylation, being able to provide data as to the position and the extent (or stoichiometry) of the modification [1–3]. Tandem mass spectrometry, wherein precursor ions are induced to dissociate using a variety of activation techniques prior to analysis of the resultant products, is the key step for localization of the modification site. Despite the advantages and availability of electron-driven techniques such as electron transfer and electron capture dissociation (ETD and ECD) [4–8], collision-induced dissociation (CID) remains the most widely used strategy for phosphorylation site mapping.

Serine and threonine phosphorylated peptides usually undergo elimination of phosphoric acid (H_3_PO_4_) or metaphosphoric acid (HPO_3_) [4] under typical low-energy CID conditions. This behavior represents a major challenge, not only to the identification of the phosphorylation site, but also for peptide sequence identification; such predominant loss of the phosphate group limits the formation of sequence specific b and y ions necessary for confident sequence identification and notably for site localization [4]. Under a low proton mobility environment, when the ionizing protons are sequestered to the most basic residues of a tryptic peptide (arginine and lysine), the elimination of (meta) phosphoric acid is thought to be promoted by the occurrence of an intramolecular hydrogen bond between the protonated *C*-terminal lysine or arginine and the oxygens of the phosphate group [9,10]. This interaction lowers the activation energy associated with the proton transfer from the *C*-terminus to the phosphate [10], thus assisting the elimination of H_3_PO_4_ via intramolecular nucleophilic substitution [9,10]. In an analogous manner to phosphomonoester-containing peptides, peptides with phosphate covalently bound to other amino acids, such as phosphohistidine (pHis), phospholysine (pLys) or phosphoarginine (pArg), are also prone to undergo prevalent neutral loss during CID [11–14]. Additionally, the mass spectrometric (MS) analysis of such phosphopeptides is made even more challenging due to the intrinsic instability of the phosphoramidate bond under the acidic conditions typically required for positive ion mode mass spectrometry [11,15], thus adding yet another hurdle to their identification by MS.

Phosphorylated peptides have been demonstrated to form non-covalent complexes (NCX) due to interaction of the phosphorylated residue with a protonated quaternary amine [16–19]. The guanidinium group of a protonated arginine (PA = 251.2 kcal/mol) [20], and the *ɛ*-ammonium group of a protonated lysine (PA = 239.4 kcal/mol) [20], can engage in strong electrostatic interactions with the phosphate of a phosphorylated amino acid residue which holds a partial or net negative charge [16]. Lone pairs of electrons on the oxygens of the *O*-phosphoester can also drive the formation of hydrogen bonds with the protonated side chains of Arg and Lys, thus increasing the overall stability of the complex [9,19,21]. An in-depth description of the strength of the hydrogen bond interaction between phosphorylated residues (Ser and Tyr) and the protonated side chains of Arg and Lys was recently provided by Rapp and co-workers [22,23]. The phosphate-ammonium bond, which plays a major role in biological systems [24], is maximized in the gas phase, a medium where electrostatic interactions are known to be stronger than in solution, due to the considerably lower dielectric constant of the vacuum (*ɛ* = 1) as compared to solvents like water (*ɛ* = 80). It is therefore expected that any such electrostatic interactions will play an even bigger role in the vacuum, controlling the gas-phase ion chemistry of the species in which it is established.

Several studies have reported on the strength of the phosphate-guanidinium non-covalent bond network [16–19]. In one of the earliest reports, Jackson et al. [17] investigated the dissociation patterns of the phosphate-arginine non-covalent bond using a Ser phosphorylated peptide and an Arg-rich basic peptide as components of the NCX. When subjected to CID, two major dissociation channels were observed: disruption of the NCX with concomitant separation of the two peptides, and the formation of a new ionic product corresponding to a species which was 80 Da larger than the basic peptide. This suggested that CID resulted in cleavage between the serine oxygen and the phosphorous atom, releasing HPO_3_ which was still engaged in electrostatic interaction with the guanidinium moiety in the Arg-rich peptide, highlighting the remarkable strength of this interaction.

Evidence of phosphate relocation during phosphopeptide CID, resulting in sequence scrambling and the limited formation of non-native phosphorylated peptides have been reported in both positive [21,25–27], and negative [28,29] ion mode. However, to our knowledge there is no evidence to date of intermolecular phosphate transfer to a previously phosphorylated peptide ion, resulting in the formation of a doubly ‘phosphorylated’ CID product. Unlike Ser, Thr and Tyr which form phosphomonoesters with a single phosphate group, His can be diphosphorylated on the 1- and 3-positions of the imidazole ring [30]. The high hydrolysis energy of the phosphoramidate bond also means that pHis/pLys/pArg residues are more likely to participate in phosphate group transfer than pSer/pThr/pTyr, a necessity of the function of pHis in two-component signaling systems [31]. Here we investigate the behavior of these more reactive pHis/pLys peptides during CID.

Using the synthetic phosphorylated peptide p(FVIAFILHLVK), containing either pHis or pLys, we demonstrate that electrospray ionization (ESI) results in a pHis homodimer which, following low-energy CID in a Paul-type ion trap, generates a product ion 80 Da bigger than the precursor ion, indicative of phosphate transfer between the two components of the dimer. MS^3^ experiments on the product of the phosphate transfer confirmed its identity as a doubly ‘phosphorylated’ peptide. Although the exact nature of the bond between the additional phosphate moiety and the original singly phosphorylated peptide has not yet been ascertained, interaction with the *N*-terminus can be excluded. That a phosphate moiety can remain bound to a positively charged Arg residue upon CID of the corresponding NCX has already been reported [14–17]; here we present the first evidence to demonstrate that such phosphotransfer can occur to an already phosphorylated peptide ion.

## 2 Materials and methods

### 2.1 Peptide phosphorylation

Peptide FVIAFILHLVK (98% purity) was synthesized (Genescript) and provided as dry powder. Potassium phosphoramidate (KNH_2_PO_3_H_2_; KPA) was synthesized from phosphoryl chloride and ammonia as described previously [32]. Phosphorylation was performed in water at pH 8 by adding 25 equivalents of KPA to 1 equivalent of peptide. The reaction was left to proceed overnight at room temperature, after which the solution was diluted to a final concentration of 1 pmol/μL in CH_3_CN:H_2_O 50:50 (v/v) and used for MS analysis.

### 2.2 ESI-QIT MS/MS

Phosphorylated peptide solutions were diluted in CH_3_CN:H_2_O 1:1 (v/v) to 1 pmol/μL and directly infused into an AmaZon ion trap (Bruker) through an electrospray source, at a flow rate of 1 μL/min. Source and octopole ion guide settings were adjusted to minimize in-source dissociation. In particular, the desolvating voltage in the ESI source and the acceleration voltages in the ion funnel were adjusted to final values of 110 and 80 V (with the standard acquisition parameters being 140 and 100 V respectively). Lower values of these two potentials resulted in a reduced ion transmission. Full scan ESI-mass spectra were acquired in the 150–2000 *m*/*z* range. CID product ion mass spectra were obtained using He as the collision gas. The MS/MS fragmentation amplitude was set at 1.20 V, and ramped from 30 to 300% of the set value.

### 2.3 Traveling wave ion mobility mass spectrometry

Phosphorylated peptide solutions were diluted in CH_3_CN:H_2_O 1:1 (v/v) to 1 pmol/μL and directly infused into a Synapt G2-*Si* HDMS instrument (Waters) through a nanospray source, at a flow rate of 0.5 μL/min. The capillary, cone voltage and source temperature were typically set to 2.7 kV, 40 V and 80 °C respectively. The IM traveling wave speed was set to 630 m/s and the wave height set at its maximum 40 V. The nitrogen drift gas flow was set at 90 mL/min for all experiments. Phosphopeptide CID was induced in the transfer cell using argon collision gas at collision energy (CE) of 30 V. For analysis of the dimer, the capillary voltage was set at 1.95 kV, while the wave speed was reduced to 311 m/s. Mass spectra were processed using MassLynx V4.1 and mobilograms using DriftScope v2.1 (both Waters, UK).

## 3 Results and discussion

### 3.1 Synthesis and characterization of p[FVIAFILHLVK]

**Fig. 1:**
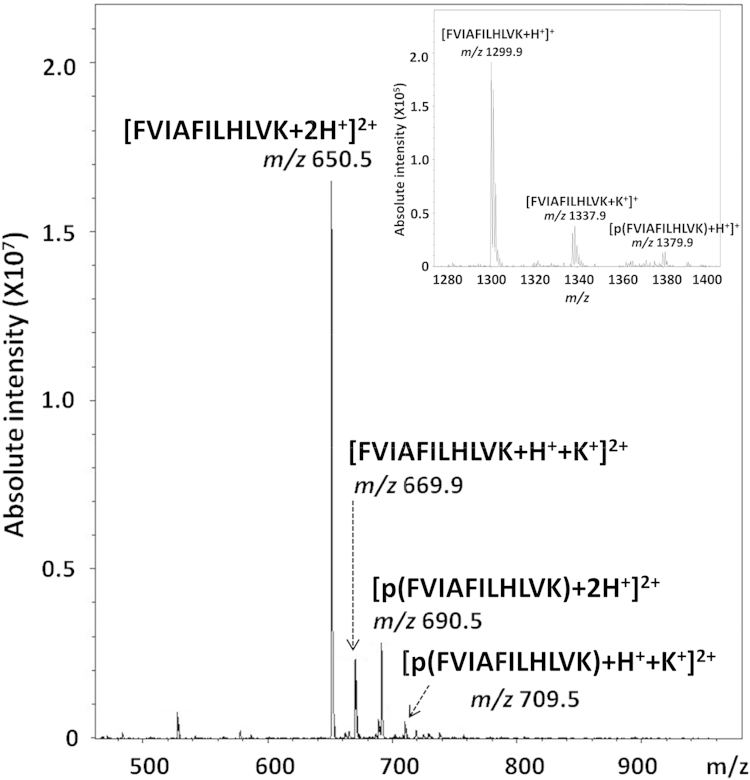
ESI-ion trap full scan mass spectrum of the products of the reaction between peptide FVIAFILHLVK and potassium phosphoramidate (KNH_2_PO_3_H_2_). Inset shows an enhanced region of the mass spectrum encompassing *m*/*z* range 1280–1400.

The products of the reaction of the non-phosphorylated synthetic peptide FVIAFILHLVK with potassium phosphoramidate (KNH_2_PO_3_H_2_; KPA) was assessed by ESI-MS/MS. The full scan ESI mass spectrum of the reaction mixture (Fig. 1) is characterized by the presence of a doubly charged ion at *m*/*z* 690.5, corresponding to the doubly protonated phosphorylated species. Also present is a doubly charged ion at *m*/*z* 650.5, matching the doubly charged non-phosphorylated FVIAFILHLVK, indicating either incomplete phosphorylation or potentially dephosphorylation of the product of the synthesis either in solution, or during ESI due to acceleration and collision with residual gas in the source region. Due to the relatively high level of potassium ions in the reaction solution, ions corresponding to the non-phosphorylated and phosphorylated peptide cationized by one proton and one potassium ion are also observed (*m*/*z* 669.9 and *m*/*z* 709.5). Singly charged ions for these species were also observed, albeit at ∼100-fold lower levels (Fig. 1 inset). Although the relative signal intensity of the non-phosphorylated species are significantly greater than the phosphorylated peptide ions, the potential difference in ionization efficiency between phosphorylated and non-phosphorylated peptides [33,34] means that no assumptions can be made regarding the relative efficiency of the reaction, save to say that it is likely to be incomplete.

**Fig. 2:**
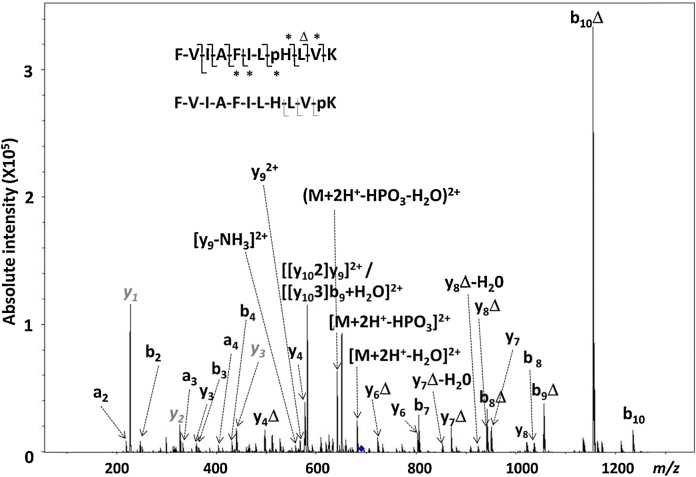
CID product ion mass spectrum of the doubly charged ion of the phosphorylated peptide p[FVIAFILHLVK+2H]^2+^ at *m*/*z* 690.5, indicating a heterogeneous population of [FVIAFILpHLVK+2H]^2+^ and [FVIAFILHLVpK+2H]^2+^, whose specific y-ions are labeled in italics (gray). (Δ) Loss of 80 Da (HPO_3_); (*) Observation of both phosphorylated and non-phosphorylated product ions.

When isolated in the ion trap and induced to dissociate by resonant excitation CID, the ion at *m*/*z* 690.5 generated a product ion mass spectrum (Fig. 2) indicative of a heterogeneous population of [FVIAFILpHLVK+2H]^2+^ and [FVIAFILHLVpK+2H]^2+^; of the product ions still retaining the phosphate group and therefore of utility for unambiguous phosphosite localization, two pHis-specific b-ions (b_8_ and b_10_) were observed, as were three y-ions (y_1_, y_2_ and y_3_) suggesting a phosphorylated *C*-terminal Lys. KPA treatment is known to phosphorylate Lys residues [35], although treatment of His-containing peptides additionally containing Lys has been reported to result preferentially in His modification, with minimal modification of Lys [36]. Interestingly, a relatively high intensity ion at *m*/*z* 581.8 was also observed (Fig. 2) which could only be ascribed to one of two products of a rearranged y-ion; [[y_10_2]y_9_]^2+^ and [[y_10_3]b_9_+H_2_O]^2+^ [37] are isobaric and cannot therefore be distinguished under these conditions. The formation of sequence scrambled products arising from y-ions, resulting in peptide sequence rearrangement, has already been reported and appears to be reliant on the presence of a basic residue near the peptide *C*-terminus, suggesting that this product is likely to have arisen from the pHis, rather than the pLys, phosphoisomer [38].

Separation of the ion at *m*/*z* 690.5 by traveling wave ion mobility-mass spectrometry (TWIMS) into two species of distinct conformation confirmed synthesis of the two phosphoisomers; the observation of pHis specific product ions deriving from the species with lower mobility (longer drift time) and pLys specific product ions from the ion with higher mobility (faster drift time) (Supp. Fig. 1) precluded the formation of the pLys-containing peptide purely as a result of gas-phase interconversion of these phosphoisomers during CID. Additionally, the formation of only singly, rather than multiply phosphorylated peptides (Fig. 1) indicates firstly that under the conditions used no di-phosphorylated His was formed (or at least was stable for analysis) and also that modification of the His and Lys residue on this peptide are mutually exclusive.

As expected, the spectrum showed intense neutral loss product ions, including at *m*/*z* 641.5 and 650.5, corresponding respectively to the elimination of HPO_3_ with or without H_2_O from the precursor ion.

### 3.2 Evidence for gas-phase phosphate transfer within the [FVIAFILpHLVK+2H^+^]^2+^dimer

**Fig. 3:**
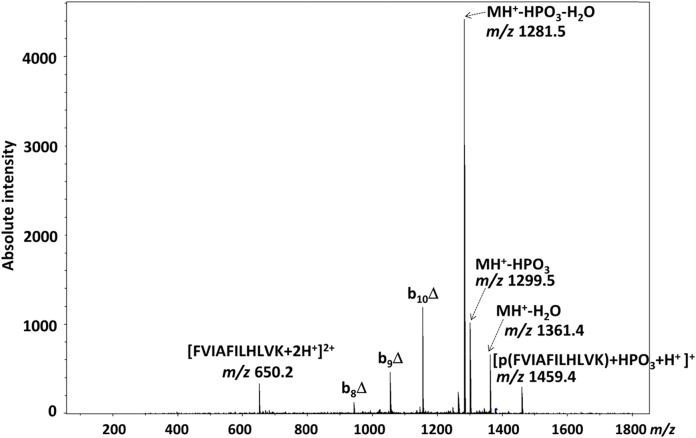
CID product ion mass spectrum of the singly charged ion of the phosphorylated peptide [p(FVIAFILHLVK)+H]^+^ at *m*/*z* 1379.5. (Δ) Loss of 80 Da (HPO_3_).

The mass spectrum of the phosphorylation reaction product (Fig. 1) also shows a peak centered at *m*/*z* 1380, corresponding to a singly protonated phosphorylated peptide p(FVIAFILHLVK) (Fig. 1 inset). As expected for a phosphorylated peptide under a low proton mobility environment [8], CID of this ionic species (Fig. 3) generated a product ion spectrum dominated by a base peak at *m*/*z* 1281.5, originating from neutral loss of HPO_3_ from the precursor ion, with concomitant elimination of H_2_O, possibly from the *C*-terminal carboxy group. Minor peaks associated with the elimination of HPO_3_ (*m*/*z* 1299.5), or of H_2_O (*m*/*z* 1361.4) from the precursor ions are also present. The only sequence-specific product ions are b_8_Δ (*m*/*z* 941.4), b_9_Δ (*m*/*z* 1054.4) and b_10_Δ (*m*/*z* 1153.4), none of which give an indication as to the phosphoisoform from which they were derived. However interestingly, a significant product ion at *m*/*z* 1459.4, 80 Da bigger than the precursor ion, can also be observed.

**Fig. 4:**
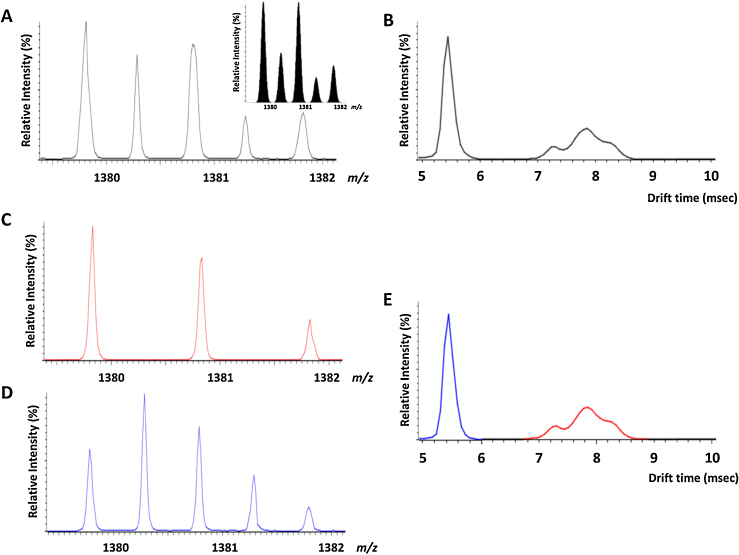
Traveling wave ion mobility-mass spectrometry analysis of the ions at *m*/*z* 1379.5 demonstrating a mixed population of singly protonated phosphopeptide monomer [M+H]^+^ and doubly protonated dimer [M_2_+2H]^2+^. (A) Isotopic distribution and (B) arrival time distribution (ATD) of the mixed population. Inset (A) depicts the theoretical isotope distribution (assuming 1:1 stoichiometry) of the [M+H]^+^ and [M_2_+2H]^2+^. Extracted ion current for the (C) longer (red) and (D) shorter (blue) ATDs (E) indicating mobility separation of the monomeric and dimeric populations.

Based on previously reported findings detailing transfer of a phosphate moiety within a NCX comprising a phosphopeptide and an Arg-containing peptide [14–17], we hypothesized that the ion at *m*/*z* 1459.4 might originate from a similar process: HPO_3_ dissociates from the pHis/pLys residue of one component of a dimer, remaining associated with the other phosphopeptide in the dimer. The original dimer [2p(FVIAFILHLVK)+2H^+^]^2+^ is isobaric to the singly protonated peptide p(FVIAFILHLVK) (*m*/*z* 1379.9) and will therefore be co-isolated prior to CID. Comparison of the experimental isotope distribution with the theoretical isotope distribution for a mixed population of the singly protonated monomer [M+H]^+^ and the doubly protonated dimer [M_2_+2H]^2+^ (Fig. 4A) certainly indicates that both species are present. Moreover, TWIMS analysis shows multiple arrival time distributions (ATDs) for these ions indicative of distinct conformations (Fig. 4B). Extraction of the ions with the longer ATD (red) yielded a mass spectrum representative of [M+H]^+^ (Fig. 4C), while the ions with the faster ATD (blue) derived from the [M_2_+2H]^2+^ species (Fig. 4D). As previously observed (Supp. Fig. 1), an important feature of the monomeric ATD (red) is the observation of multiple non-resolved conformations (Fig. 4E) corresponding (in part) to conformational differences of the pLys and pHis phosphoisomers.

Observation of a doubly charged product ion at *m*/*z* 650.2 in Fig. 3, corresponding to a doubly protonated species of the non-phosphorylated peptide FVIAFILHLVK produced via HPO_3_ elimination, further supports the theory of intermolecular phosphate transfer by means of dimer formation. If the ionic population centered at *m*/*z* 1379.5 were only composed of singly charged p(FVIAFILHLVK), the doubly charged species at *m*/*z* 650.2 could not possibly be generated. Additionally, the possibility that the dimer may be composed of a doubly phosphorylated and a non-phosphorylated peptide can be discarded on account of the fact that a doubly phosphorylated peptide could not be detected as either a singly protonated (*m*/*z* 1459.5) or doubly protonated (*m*/*z* 730.5) ion upon ESI-MS of the reaction mixture (Fig. 1).

**Fig. 5:**
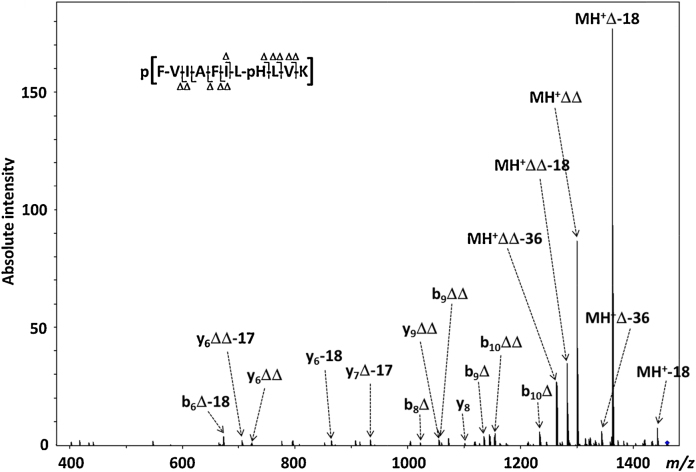
MS^3^ CID product ion mass spectrum of ions at *m*/*z* 1459.4 generated by CID of ions at *m*/*z* 1379.5. The doubly ‘phosphorylated’ peptide is represented by p(FVIAFILpHLVK). (Δ) Loss of 80 Da (HPO_3_).

To confirm our hypothesis, an MS^3^ experiment was performed in which the ion centered at *m*/*z* 1380 (corresponding to the phosphopeptide homodimer [2p(FVIAFILHLVK)+2H^+^]^2+^; Fig. 1) was subjected to CID, prior to isolation and CID of the resultant product ion at *m*/*z* 1459 (Fig. 5). The main peaks in the MS^3^ spectrum are associated with the loss of HPO_3_ + H_2_O (MH^+^Δ-18) from the precursor (*m*/*z* 1361), and elimination of two molecules of HPO_3_ (*m*/*z* 1299) with concomitant elimination of one (*m*/*z* 1281) and two (*m*/*z* 1263) molecules of H_2_O. The observation of these elimination pathways, along with the presence of some sequence specific diagnostic ions, albeit at a very low signal-to-noise level, confirmed the identity of the ionic species at *m*/*z* 1459 as that of an intact phosphorylated peptide (FVIAFILpHLVK/FVIAFILHLVpK) holding an extra HPO_3_ moiety.

**Scheme 1:**
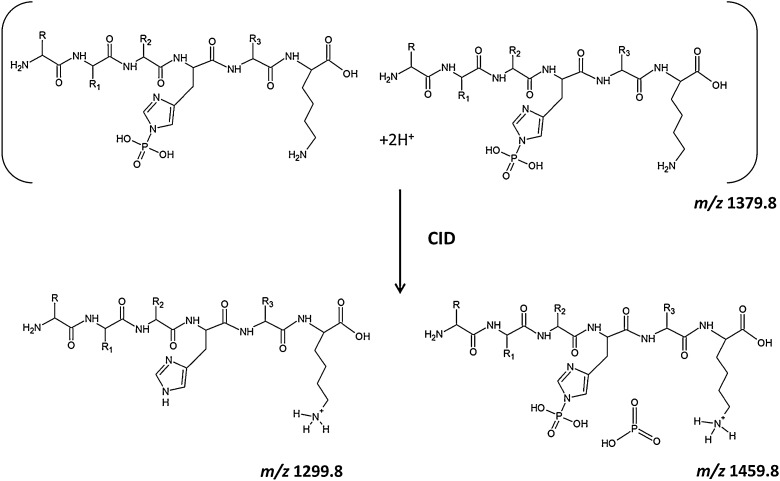
Proposed mechanism for the formation of the ions at *m*/*z* 1459.8. The scheme depicts a homodimer of the phosphopeptide FVIAFILpHLVK (*m*/*z* 1379.8), whose components can undergo elimination of metaphosphoric acid HPO_3_ and generation of a transient ternary complex, which then evolves to give the dephosphorylated peptide at *m*/*z* 1299.8 and the ‘doubly’ phosphorylated peptide at *m*/*z* 1459.8.

Based on current understanding, it is likely that the additional phosphate group is kept within the complex by means of hydrogen bonds and charge–dipole interactions with the *C*-terminal protonated Lys residue (Scheme 1). The peptide under investigation contains a basic *C*-terminal Lys and can therefore generate a protonated primary amine. Given the well-characterized interaction between a phosphate group and a protonated Lys [22], we anticipated that a similar non-covalent bond could be established between the phosphate on pHis and the ɛ-ammonium group of the *C*-terminal Lys, thus mimicking the systems previously described [16–19]. If true, this should facilitate the formation of a homodimer via electrostatic and hydrogen bond interactions. Additionally, the Π-electron density of the aromatic imidazole ring of the His and the phenyl ring of the Phe residues could be engaged in a Π-cation interaction with the protonated side chain of the lysine residue, thus contributing to the overall stability of the dimer.

Phosphate transfer was not observed upon CID of the related peptide FVIAFILpHLV (Supp. Fig. 3), generated by proteolytic cleavage of the *C*-terminal Lys residue by carboxypeptidase-B (CBP-B), demonstrating that an unmodified *C*-terminal Lys residue is required for stabilization of the homodimeric NCX and subsequent phosphate transfer. We therefore envisage a scenario in which a homodimer of the phosphorylated peptide FVIAFILpHLVK survives in the gas phase, stabilized by non-covalent interactions between its monomers.

Based on the isotopic distribution of the ionic population centered at *m*/*z* 1380, the system is likely composed of a combination of the singly charged pHis/pLys phosphorylated peptides [p(FVIAFILHLVK)+H^+^]^+^ and the doubly charged homodimer [2(FVIAFILpHLVK)+2H^+^]^2+^. When activated by CID, the singly charged phosphoramidate-containing peptide ions undergo extensive elimination of metaphosphoric acid (with concomitant elimination of H_2_O), to yield the corresponding neutral loss product ions (*m*/*z* 1299 and *m*/*z* 1281 respectively), with limited formation of sequence-specific product ions. The pHis homodimer, on the other hand, is likely to dissociate to yield its respective monomers, with one of them undergoing elimination of HPO_3_, thus appearing on the spectrum at *m*/*z* 1299 (singly charged) and *m*/*z* 650.2 (doubly charged). Release of HPO_3_ from one of the monomers is accompanied by capture of the metaphosphate by the other monomer likely in a concerted mechanism [14–17], leading to the doubly phosphorylated species at *m*/*z* 1459 (Scheme 1). Although the exact nature of the interaction between HPO_3_ and the phosphorylated monomer cannot be unambiguously defined based on our data, the strength of this bond is such that it can survive the CID process, thereby generating the ionic species at *m*/*z* 1459. Interestingly, only the dephosphorylated peptide is released upon dissociation of the dimer, appearing as a doubly charged ion at *m*/*z* 650.2 (Fig. 3), suggesting that the whole ionic population corresponding to the phosphorylated peptide is involved in the formation of the doubly phosphorylated species. Observation of the y_8_ and y_6_-18 ions in the MS^3^ spectrum that retain both phosphate groups, indicates that the second phosphate group is stabilized within the *C*-terminal region of the peptide as would be expected, although the exact site cannot be localized. Gas-phase relocation of metaphosphate from phosphorylated Ser, Thr and Tyr residues to the *C*-terminal carboxyl group, and even to amide groups, has been reported upon the CID of deprotonated phosphorylated peptides [28,29]. Transfer of HPO_3_ to the *C*-terminal carboxy group could be envisaged in this case. However, the observed loss of two molecules of HPO_3_ from b_9_ and b_10_ product ions in Fig. 5 points toward His as the most likely acceptor residue in the structure of the ions at *m*/*z* 1459.

## 4 Conclusions

Confirming what has previously been reported for NCXs between phosphorylated peptides and Arg-rich sequences, the present work reports for the first time on a homodimeric NCX between identical pHis-containing peptides. The driving force for the formation and survival of the homodimer in the gas phase is likely provided by electrostatic interaction between the phosphates and protonated side chain of the *C*-terminal Lys residue, with which the phosphates also form hydrogen bond interactions. The strength of the non-covalent bonds between the pHis and the protonated *C*-terminal Lys is reflected by the peculiar dissociation behavior of the gas-phase dimer, which results in the transfer of a phosphate group from one phosphopeptide monomer to the other, leading to the observed doubly phosphorylated peptide ion.
