# Supplementary material for: Gas-phase intermolecular phosphate transfer within a phosphohistidine phosphopeptide dimer
Source: Int J Mass Spectrom. 2014 Jun 15;367:28–34. doi: 10.1016/j.ijms.2014.04.015 (PMC4375673; doi:10.1016/j.ijms.2014.04.015)
Supplement: Supplementary file 1 [file mmc1.docx]

**Supplementary Figure 1.** Mobility separation of (A) the doubly charged phosphorylated peptide ion at *m/z* 690.4 and the extracted drift time (bins) for the phospholysine (pLys)-specific y_1_ product ion at *m/z* 227.1 (B) and the phosphohistidine (pHis)-specific b_8_^2+^ product ion at *m/z* 511.3 (C).


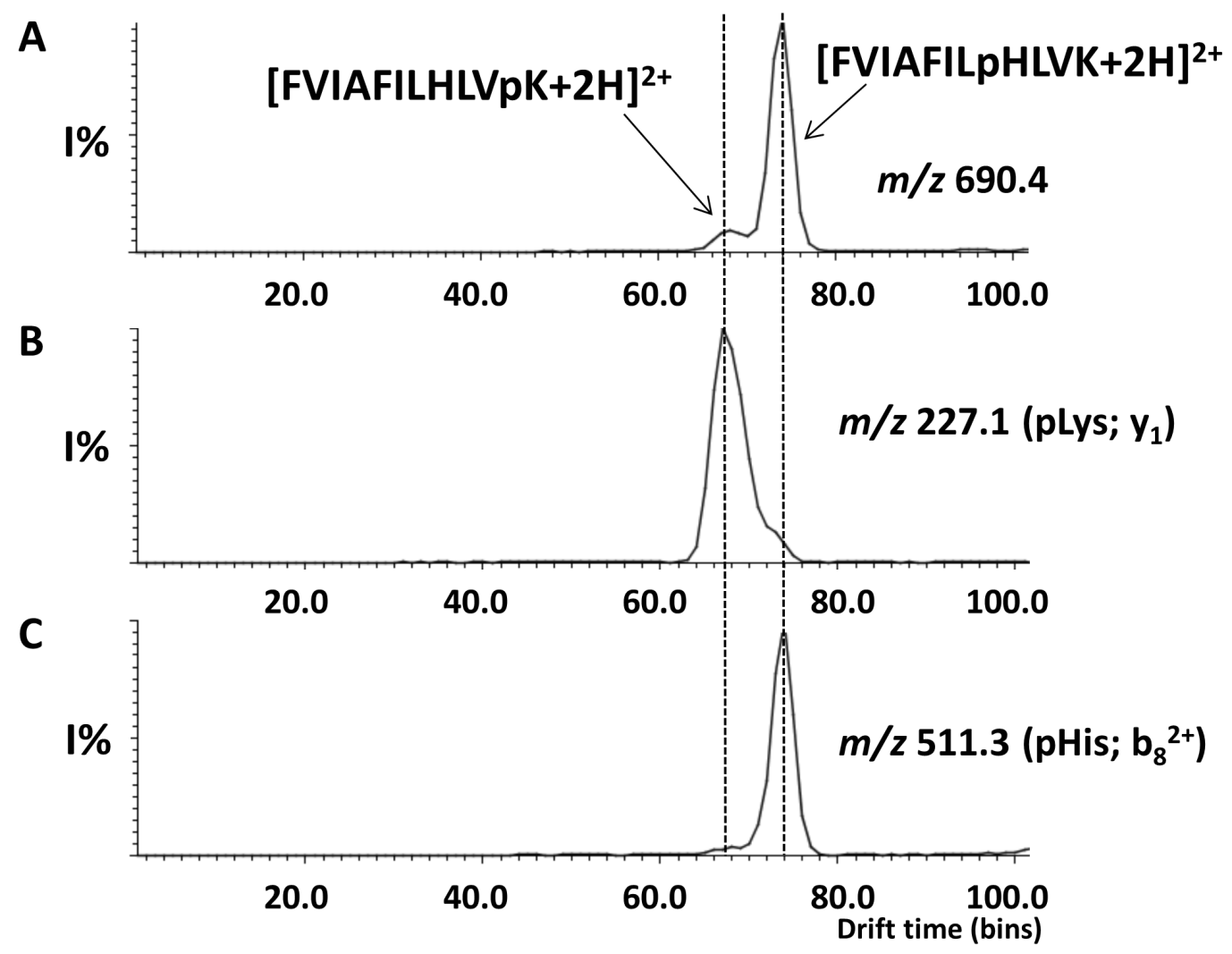


**Supplementary Figure 2.** CID product ion mass spectrum of the doubly charged ion of the phosphorylated peptide [p(FVIAFILHLVK) + 2H]^2+^ at *m/z* 690.5 acquired on the SYNAPT G2-S*i* HDMS, indicating a heterogeneous population of [FVIAFILpHLVK + 2H]^2+^ and [FVIAFILHLVpK + 2H]^2+^, whose specific y-ions ions are labeled in italics (grey). Δ indicates loss of 80 Da (HPO_3_).


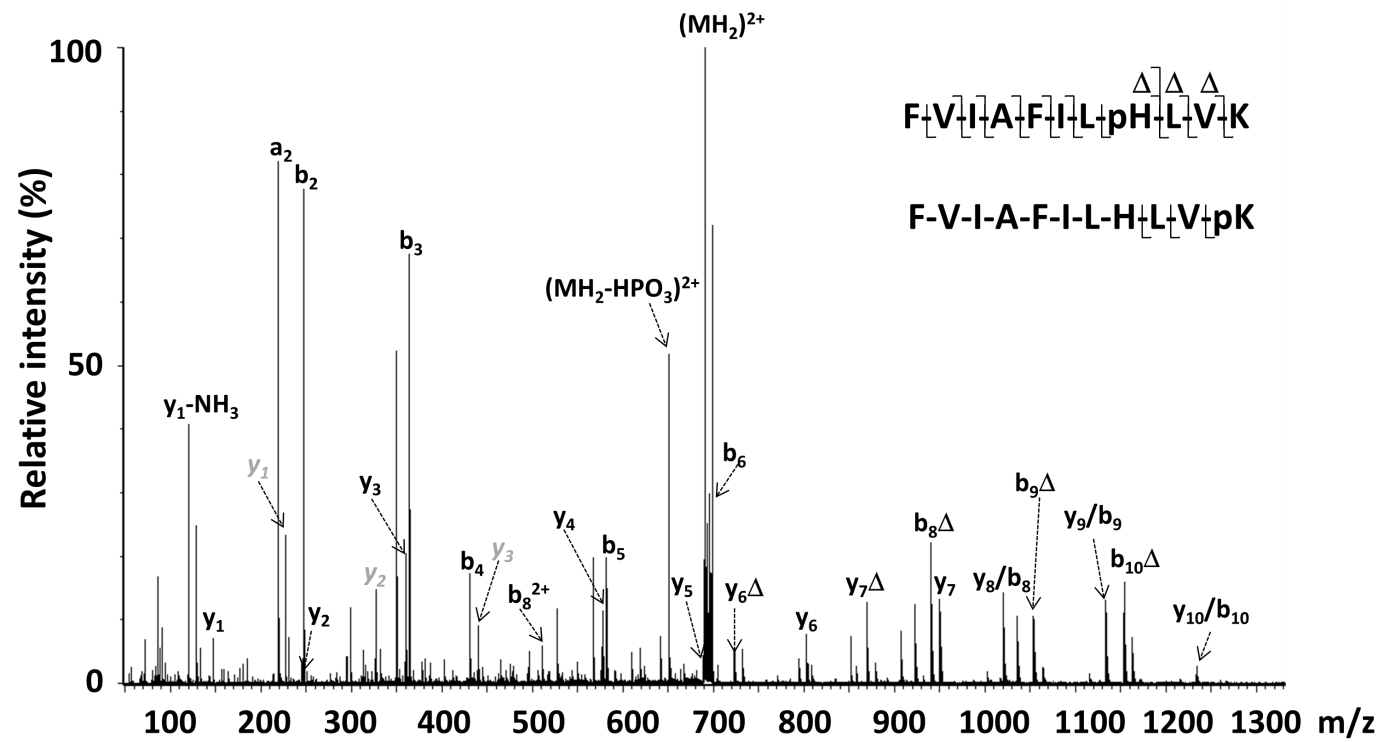


**Supplementary Figure 3.** CID product ion mass spectrum of the singly charged ion of the carboxypeptidase-B treated phosphorylated peptide [FVIAFILpHLV + H]^+^ at *m*/z 1251.6. Δ indicates loss of 80 Da (HPO_3_).

**
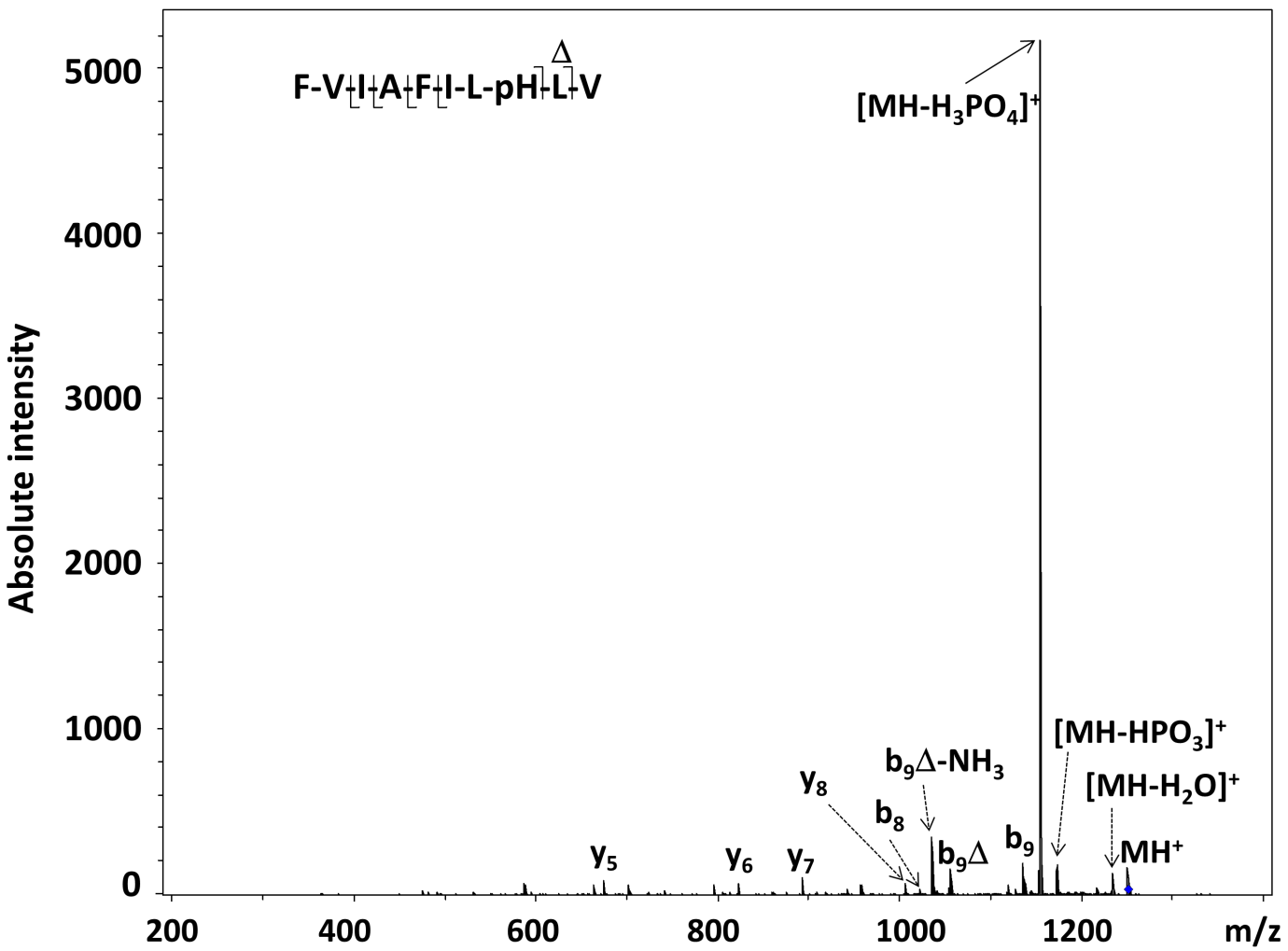
**
